# Evolution of Anti-citrullinated Protein Antibodies in Rheumatoid Arthritis Patients Under Disease-Modifying Therapy: A Prospective Cohort Study

**DOI:** 10.7759/cureus.80217

**Published:** 2025-03-07

**Authors:** Chaimae Charoui, Bouchra Amine, Imane El Binoune, Salma Zemrani, Samira Rostom, Amal Bouziane, Rachid Bahiri

**Affiliations:** 1 Rheumatology A, El Ayachi Hospital, Ibn Sina University Hospital, University Mohamed V, Rabat, MAR; 2 Laboratory of Biostatistics, Clinical Research and Epidemiology, University Mohamed V, Rabat, MAR

**Keywords:** anti-citrullinated protein antibodies, disease activity score (das-28), immunological remission, rheumatoid arthritis, rituximab

## Abstract

Introduction

The primary objective of our study was to describe the variation of anti-citrullinated protein antibodies (ACPA) antibody levels in patients with rheumatoid arthritis (RA) under disease-modifying therapy. The secondary objectives were to evaluate the variation of ACPA levels according to disease activity and to determine the characteristics of patients who have negative ACPA.

Methods

It was a prospective study including patients with ACPA-positive RA treated with disease-modifying antirheumatic drugs (DMARDs) and having a Disease Activity Score (DAS) greater than 2.6, indicating at least low disease activity or higher. A medical checkup including a clinical evaluation, an inflammatory test, and a measurement of ACPA levels using the enzyme-linked immunosorbent assay (ELISA) was performed for each patient at M0, M6, and M12. Two groups were defined: the rituximab (RTX) group, consisting of patients treated with RTX, and the no-RTX group, which included patients receiving other therapies, such as tumor necrosis factor inhibitors (TNFi) and interleukin 6 inhibitors (anti-IL6) or conventional disease-modifying antirheumatic drugs (csDMARDs).

Results

Ninety ACPA-positive RA patients were included. Seventy-seven (85.6%) were female and the mean age was 54.96 ± 13.13 years. The median disease duration was 11 years, and the baseline Disease Activity Score 28-C-reactive protein (DAS28-CRP) was 4.35 ± 0.99. Forty-eight (53.3%) of patients were already on RTX. The median ACPA level at baseline was 186 IU/ml (74-200), showing a significant decline over time (M0: 186 (74-200); M6: 111 (59-195); M12: 95 (45-195); p<0.001). This reduction was significant in the RTX group (ACPA M0: 191 (70-200); ACPA M6: 96 (33-195); ACPA M12: 75 (17-147); p<0.001) but not in the no-RTX group (ACPA M0: 156 (68-200); ACPA M6: 121 (70-195); ACPA M12: 175 (67-200); p=0.26).

At M12, 27 (30%) patients achieved remission based on the DAS28 score. Their median ACPA level was 157 IU/ml (75-200) at baseline, which significantly decreased over time (p<0.001). When analyzing the delta ACPA, it was significantly larger in the RTX group (p<0.001).

Conclusion

In this real-life study, ACPA antibody levels decreased significantly in RA patients who had received RTX. This could suggest the possibility of achieving immunological remission with biotherapy, more specifically with RTX.

## Introduction

Rheumatoid arthritis (RA) is the most common chronic inflammatory rheumatic disease. It mainly affects women, with peak incidence between 40 and 60 years old [1].

Immunologically, there are two main subtypes of RA, defined by the presence or absence of anti-citrullinated protein antibodies (ACPA) and/or rheumatoid factor (RF) [2].

ACPAs are detected in around two-thirds of patients with RA, with very good diagnostic specificity of 90%, and help in the diagnosis of early and undifferentiated arthritis [3]. However, the pathophysiological role of ACPA in the development or progression of RA is still not well-defined. According to studies, ACPA-positive patients have a more aggressive clinical phenotype than ACPA-negative patients [4]. Also, it was found that a high concentration of ACPA predicts worse disease activity and faster radiographic progression [5]. 

In terms of treatment response, studies indicate that ACPA-negative patients tend to have a less favorable response to therapies such as rituximab (RTX) [6].

This effect is explained by the fact that RTX primarily targets B cells, inhibiting the differentiation of plasmablasts into plasmocytes and consequently reducing total immunoglobulin (Ig) production. As a result, serum biomarkers of B-cell activation, such as rheumatoid factor, ACPA, or elevated IgG levels, have been identified as potential markers for predicting RTX response [7].

Currently, clinical, biological, and ultrasound remission are the primary treatment goals in RA [8]. However, the concept of immunological remission remains a subject of debate. The existing literature on the definition of immunological remission is still evolving and the literature data concerning the decrease in ACPA levels under disease-modifying therapies is still contradictory.

The primary objective of our study was to describe the variation of ACPA antibody levels in patients with RA under disease-modifying therapy. The secondary objectives were to evaluate the variation of ACPA levels according to disease activity and determine the characteristics of patients with negative ACPA.

## Materials and methods

Study design and setting

We conducted a prospective cohort study including patients with RA according to the American College of Rheumatology/European League Against Rheumatism (ACR/EULAR) 2010 classification criteria [6]. Patients were recruited from the Rheumatology Department at El Ayachi University Hospital in Rabat between May 2022 and October 2022. We followed the Strengthening the Reporting of Observational Studies in Epidemiology (STROBE) guidelines for cross-sectional studies in the preparation of our report [9].

Participants

Eligible participants were older than 18 years old with RA. At baseline (M0), patients were already receiving biological disease-modifying antirheumatic drugs (bDMARDs) or conventional synthetic disease-modifying antirheumatic drugs (csDMARDs), and had a Disease Activity Score (DAS) greater than 2.6, indicating at least low disease activity or higher. Patients with ACPA-negative RA or ACPA-positive RA in remission were excluded from the study. 

Participants were categorized into groups based on their treatment. Two groups were defined: the RTX group, consisting of patients treated with RTX, and the no-RTX group, which included patients receiving other therapies, such as tumor necrosis factor inhibitors (TNFi) and interleukin 6 inhibitors (anti-IL6) or csDMARDs. Follow-up visits were conducted at three time points: baseline (M0), six months (M6), and 12 months after the start of the study (M12).

Regarding the RTX treatment regimen at our institution, rituximab is not administered on a fixed schedule but is tailored to the individual patient’s disease activity. It is typically given when there is a significant flare-up or exacerbation of disease activity, rather than on a set schedule like every six or 12 months. 

It is important to highlight that participants in this study were already receiving biologic therapies prior to the start of the study, and their treatment regimens remained unchanged during the study period. Also, in the RTX group, the latest RTX infusion was given over a year before enrollment.

Variables and outcomes

The primary outcome of the study was the variation in ACPA levels over time according to treatment groups. Secondary outcomes included the assessment of ACPA variation over time based on disease activity levels and the identification of patient characteristics associated with ACPA negativation. Regarding disease activity at M12, three groups were defined: the remission group; the low disease activity group (LDA); and the active disease group, where the DAS28 score was greater than 3.2.

The main exposure of interest was the type of treatment received: RTX versus no-RTX treatments (including TNFi, anti-IL-6 and csDMARDs).

Data collection

Data collected included socio-demographic information (age, sex, family history of RA, disease duration, and comorbidities) and clinical characteristics. Clinical and para-clinical data were evaluated using DAS28 score, erythrocyte sedimentation rate (ESR), and C-reactive protein (CRP) levels. ACPA levels were measured at each time point (M0, M6, and M12) in the same laboratory using enzyme-linked immunosorbent assay (ELISA), with a cutoff value set at 20. Medication use, including past and current treatments with DMARDs, and corticosteroid therapy was also documented.

Ethics approval

The study protocol was reviewed and approved by our local institutional review boards and the national ethics committee, the Ethics Committee for Biomedical Research at Mohammed V University in Rabat, Faculty of Medicine and Pharmacy of Rabat. The committee's reference number is CERB 118-24.

Sampling and statistical analysis 

The study included all patients who were regularly followed up at the day hospital during the recruitment period.

Statistical analysis was conducted using SPSS (IBM Corp., Armonk, NY, USA). Qualitative data frequencies were presented as both numbers and percentages. The mean ± standard deviation was used for parameters with a normal distribution, and the median ± interquartile range (IQR) for those with an asymmetric distribution.

The comparison of ACPA titers between M0, M6 and M12 in each of the treatment groups was performed using the Friedman test. The delta ACPA was evaluated between the RTX and no-RTX groups using the Mann-Whitney U test. Additionally, comparisons of delta ACPA between groups, according to disease activity, were conducted using the Kruskal-Wallis test. The significance level was set at p<0.05.

## Results

Patient characteristics

Ninety patients with ACPA-positive RA were included in the study. Social characteristics, disease and treatment characteristics are shown in Table 1. Seventy-seven (85.6%) were female. The mean age was 54.96 years ± 13.13. The median disease duration of RA was 11 years (7-16). The mean DAS28 CRP score at baseline was 4.35 ± 0.99. Forty-eight (53.3%) were on RTX. Eighty (89%) patients were on corticosteroid therapy.

**Table 1:** Sociodemographic, clinical and paraclinical characteristics of the study population according to treatment groups at inclusion RTX: Rituximab; LAA: atloidoaxial luxation; DAS: Disease Activity Score; ESR: erythrocyte sedimentation rate; CRP: C-reactive protein; ACPA: anti-citrullinated protein antibodies; csDMARDS: Conventional Disease Modifying Antirheumatic Drugs

Variables	Total (n=90)	RTX group (n=48)	No-RTX group (n=42)		p
Age, years, mean±SD	54.96±13.13	54.90±14.1	55.02±12.23	0.96	
Female, n(%)	77(85.6)	42(87.5)	35(83.3)	0.57	
Disease duration, years, median (IQR)	11(7-15)	11.5(7.25-14)	10(6.5-16)	0.93	
Onset mode (Polyarticular) n(%)	78(86.67)	42(87.5)	36(85.7)	0.42	
Erosive character, n(%)	71(78.9)	39(81.3)	32(76.2)	0.28	
LAA, n(%)	7(7.8)	3(6.3)	4(9.5)	0.70	
Interstitial lung disease, n(%)	17(18.9)	12(25)	5(11.9)	0.17	
DAS28-ESR, mean±SD	5.13±1.18	5.20±1.20	5.04±1.18	0.54	
DAS28CRP, mean±SD	4.35±0.99	4.45±1	4.25±0.97	0.34	
ACPA, median (IQR)	186(74-200)	190(71-200)	164(73-200)	0.81	
Corticotherapy, n(%)	71(78.9)	40(83.33)	31(73.8)	0.69	
csDMARDs, n(%)	61(67.78)	34(70.83)	27 (64.28)	0.44	

The clinical and paraclinical characteristics were comparable between the RTX and no-RTX groups (Table 1). Also, when comparing baseline characteristics between the three groups defined according to disease activity at M12, no significant differences were observed.

Changes in ACPA levels according to treatment group

After RTX treatment, the decrease in ACPA levels between M0 and M6 and M12 was significant with p<0.001; (ACPA M0: 191 (70-200); ACPA M6: 96 (33-195); ACPA M12: 75 (17-147); p<0.001). However, it was not significant in the group that did not receive RTX (p=0.26) (Table 2).

**Table 2:** Comparison of ACPA levels between M0, M6 and M12 according to treatment group RTX: Rituximab; ACPA: Anti-citrullinated Peptide Antibody

Variables	ACPA M0, median (IQR)	ACPA M6, median (IQR)	ACPA M12, median (IQR)		p
N (90)	186(70-200)	111(59-195)	95(45-195)	<0.001	
RTX group (n=48)	191(70-200)	96(33-195)	75(17-147)	<0.001	
No-RTX group (n=42)	156(68-200)	121(70-195)	175(67-200)	0.26	

Comparing the two groups, RTX and no-RTX, the δACPA was significantly larger in the RTX group (p<0.001) (Table 3).

**Table 3:** Comparison of δACPA between different treatment groups RTX: Rituximab; ACPA: Anti-citrullinated Peptide Antibody

Variables	RTX group (n=48)	No-RTX group (n=42)	p
Delta ACPA M6.M0,median (IQR)	-18 (-94- -5)	-4(-39-35)	0.01
Delta ACPA M12.M6, median (IQR)	-16 (-70- -5)	0(-11.25-16.02)	<0.001
Delta ACPA M12.M0, median (IQR)	-60 (-105- -23)	-0.5(-56- 56)	<0.001

Changes in ACPA according to disease activity

Twenty-seven (30%) patients achieved DAS28 remission at M12, of whom 16 (59%) were on RTX, nine (33%) on TNFi, one (4%) on anti-IL6 and one (4%) on csDMARDs.

In the group that achieved remission at M12, the median ACPA level was 157 IU/ml (75-200) at baseline. A significant decrease in ACPA levels was noted (ACPA M0: 157 (75-200); ACPA M6: 95 (50-175); ACPA M12: 95 (29-195); p<0.001). However, a significant decrease in ACPA level was also observed in the group that remained active at M12 (Table 4).

**Table 4:** Comparison of ACPA levels between M0, M6 and M12 according to disease activity at M12 LDA: low disease activity; Gr: group; ACPA: Anti-citrullinated Peptide Antibody

Variables	ACPA M0, median (IQR)	ACPA M6, median (IQR)	ACPA M12, median (IQR)		p
Gr remission at M12(n=27)	157(75-200)	95(50-175)	95(29-195)	<0.001	
Gr LDA at M12(n=17)	168(64-200)	170(43-195)	110(71-180)	0.14	
Gr active at M12(n=46)	195(72-200)	167(69-195)	170(69-195)	0.001	

There was no significant difference in δACPA between the different groups (remission, LDA and active) (Table 5).

**Table 5:** Comparison of δACPA between subgroups according to disease activity at M12 LDA: low disease activity; Gr: group; ACPA: Anti-citrullinated Peptide Antibody

Variables	Gr remission M12 (n=27)	Gr LDA M12 (n=17)	Gr active M12 (n=46)	p
Delta ACPA M6.M0, median (IQR)	-25(-95- -5)	-8(-98-4)	-14(-45-11)	0.32
Delta ACPA M12.M6, median (IQR)	-4(-27- 16)	-12(-22.5- -2.25)	-10(-29- 5)	0.57
Delta ACPA M12.M0, median (IQR)	-31(-110- 5)	-23(-105- 12)	25(-96- 0)	0.88

Negativation of ACPA

Despite the decrease in ACPA levels under biological treatment, none of the patients tested negative for ACPA antibodies at M12.

## Discussion

In this study, we observed that ACPA antibody levels decreased significantly in RA patients who had received RTX. We also noted that the decrease of ACPA was significant in the group that achieved remission at M12 but also in the group that remained active. However, at M12, none of the patients tested negative for ACPA.

In our context, RTX is by far the most prescribed biological in Morocco given its availability in hospitals and the endemic tuberculosis in the country. Even after the COVID-19 pandemic, RTX continues to be favored primarily due to its lower cost. However, its use requires careful precautions and regular monitoring to ensure patient safety. A study conducted using data from the Moroccan Biotherapy Registry (RBSMR) including 225 patients with RA found that RTX was the first bDMARD prescribed [10].

Also, RTX's interference with the immune system, particularly its effect on B cells, led us to compare the variation in ACPA levels between the RTX and no-RTX groups.

On the question of variations in ACPA under treatment, a study conducted in the rheumatology departments of three hospitals in the Paris region evaluated changes in ACPA levels under abatacept, RTX and TNFi found similar results and suggested a specific effect of RTX on the reduction of ACPA antibody levels [11].

In addition, in a prospective non-randomized observational study including 100 RA patients, the primary aim was to analyze the effect of biological DMARD therapy on anti-cyclic citrullinated peptide 2 antibody levels over two years, levels significantly decreased in patients receiving RTX and abatacept, while they remained basically unchanged in patients receiving continuous treatment with methotrexate, TNFi or anti-IL6 [12].

These findings suggest that ACPA autoantibody responses may be attenuated in some RA patients, particularly if DMARDs that interfere with the adaptive immune response are used [13].

Based on the hypothesis of a direct pathogenic role of ACPAs, it can be suggested that a significant reduction or even disappearance of ACPA titres, particularly at the start of the disease, could be important in reducing structural damage in RA [14].

Nevertheless, the immunological remission in RA is not well defined and the results are contradictory [15]. Also, there is limited data on the relationship between changes in ACPA levels and disease activity. The impact of these changes has not been extensively studied [16].

In a US observational cohort including 840 RA patients, the aim was to assess associations between changes in ACPA levels and disease activity in patients with RA. This study indicates that reductions in ACPA levels are associated with a reduction of disease activity and increased clinical benefit [17].

Another retrospective study including 143 patients showed a significant decrease in ACPA levels and RF after six months of treatment. This decrease was also significantly associated with a reduction in disease activity, while in the non-responders, the antibody levels did not decline significantly [18].

However, a prospective study including 381 RA patients taking part in the IMPROVED trial showed that changes in RA autoantibody levels did not correlate with clinical activity or response to long-term treatment [16].

Another long-term observational study aimed to determine whether autoantibodies disappeared in RA patients who achieved sustained remission without DMARDs and concluded that disappearance of autoantibodies rarely occurred and that patients who achieved prolonged remission of RA did not certainly become seronegative [19]. These results are consistent with our findings.

One limitation of this study is the sample size, as participants were recruited from a single tertiary center, which may limit the generalizability of the findings. Furthermore, it is important to note that all patients were already receiving biologic therapy before enrollment. Specifically, in the RTX group, the most recent rituximab infusion had taken place at least one year prior to enrollment. This extended time interval may have diminished the direct effect of rituximab on ACPA levels observed at baseline, potentially limiting its influence on the markers at the time of study inclusion. Although we were able to show significant results by following this population, larger studies are needed in the future to establish the correlation between the reduction in ACPA levels and clinical remission.

## Conclusions

In this real-life study, ACPA antibody levels decreased significantly in RA patients who had received RTX. This could suggest the possibility of achieving immunological remission with biotherapy, more specifically with RTX. However, immunological remission needs further studies to be properly defined.
